# Supplementary material for: 19-(Benzyloxy)-19-oxojolkinolide B (19-BJB), an ent-abietane diterpene diepoxide, inhibits the growth of bladder cancer T24 cells through DNA damage
Source: PLoS One. 2021 Mar 16;16(3):e0248468. doi: 10.1371/journal.pone.0248468 (PMC7963099; doi:10.1371/journal.pone.0248468)
Supplement: S4 Fig — (DOCX) [file pone.0248468.s005.docx]

**S4 Fig. Plot of actual activity against predicted activity by the CoMFA model.**
