# Supplementary material for: 19-(Benzyloxy)-19-oxojolkinolide B (19-BJB), an ent-abietane diterpene diepoxide, inhibits the growth of bladder cancer T24 cells through DNA damage
Source: PLoS One. 2021 Mar 16;16(3):e0248468. doi: 10.1371/journal.pone.0248468 (PMC7963099; doi:10.1371/journal.pone.0248468)
Supplement: S1 Table — (DOCX) [file pone.0248468.s007.docx]

**S1 Table. Statistical parameters of CoMFA model.**

| **Statistical parameters** | **Value** |
| --- | --- |
| R^2^_LOO_*^a^* | 0.689 |
| R^2^_CV_*^b^* | 0.706 |
| ONC_LOO_*^c^* | 1 |
| ONC_CV_*^d^* | 1 |
| SEP_LOO_*^e^* | 0.377 |
| SEP_CV_*^e^* | 0.367 |
| SEE*^f^* | 0.158 |
| F-test ratio*^g^* | 90.564 (6, 26) |
| R^2^*^h^* | 0.954 |
| Steric*^i^* contribution | 0.501 |
| Electrostatic*^j^* contribution | 0.499 |

*^a^*R^2^_LOO_ = cross-validated correlation coefficient from LOO. *^b^*R^2^_cv_ = cross-validated. *^c^*ONC_LOO_ = optimal number of components. *^d^*ONC_CV_ = optimal number of components. *^e^*SEP = standard error of prediction. *^f^*SEE = standard error of estimate. *^g^*F-test ratio = r^2^/(1-r^2^). ***^i^***Steric = percentage of field contribution about steric. ***^j^***Electrostatic = percentage of field contribution about electrostatic.
