# Supplementary material for: 19-(Benzyloxy)-19-oxojolkinolide B (19-BJB), an ent-abietane diterpene diepoxide, inhibits the growth of bladder cancer T24 cells through DNA damage
Source: PLoS One. 2021 Mar 16;16(3):e0248468. doi: 10.1371/journal.pone.0248468 (PMC7963099; doi:10.1371/journal.pone.0248468)
Supplement: S2 Table — (DOCX) [file pone.0248468.s008.docx]

**S2 Table. The actual and predicted pIC_50_ values of the jolkinolide derivatives.**

|  | **IC_50_ (μM)** | **pIC_50_^a^ (M)** | **Predicted pIC_50_ (M)** | **Residual** | **Molecular Weight** |
| --- | --- | --- | --- | --- | --- |
| JA00 | 88.43 | 4.0534 | 3.933 | 0.1204 | 314 |
| JA01 | 345.8 | 3.4612 | 3.579 | -0.1178 | 344 |
| JA02 | 26.16 | 4.5824 | 4.594 | -0.0116 | 434 |
| JA03 | 193.5 | 3.7133 | 3.61 | 0.1033 | 358 |
| JA04 | 132.8 | 3.8768 | 3.945 | -0.0682 | 372 |
| JA05 | 45.53 | 4.3417 | 4.159 | 0.1827 | 386 |
| JA06 | 49.66 | 4.304 | 4.262 | 0.042 | 400 |
| JA07 | 417.8 | 3.379 | 3.638 | -0.259 | 330 |
| JA08 | 32.11 | 4.4934 | 4.583 | -0.0896 | 313 |
| JA09 | 114.4 | 3.9416 | 3.876 | 0.0656 | 484 |
| JA10 | 82.83 | 4.0818 | 4.101 | -0.0192 | 372 |
| JA11 | 38.03 | 4.4199 | 4.49 | -0.0701 | 386 |
| JA12 | 27.41 | 4.5621 | 4.409 | 0.1531 | 434 |
| JA13 | 318.8 | 3.4965 | 3.582 | -0.0855 | 346 |
| JA14 | 180.3 | 3.744 | 3.817 | -0.073 | 430 |
| JA15 | 25.70 | 4.5901 | 4.534 | 0.0561 | 458 |
| JA16 | 16.57 | 4.7807 | 4.72 | 0.0607 | 554 |
| JB00 | 23.00 | 4.6383 | 4.798 | -0.1597 | 330 |
| JB01 (19-BJB) | 2.165 | 5.6645 | 5.671 | -0.0065 | 450 |
| JB02 | 5.766 | 5.2391 | 5.192 | 0.0471 | 374 |
| JB03 | 14.52 | 4.8386 | 4.913 | -0.0744 | 388 |
| JB04 | 8.451 | 5.0731 | 4.853 | 0.2201 | 402 |
| JB05 | 31.98 | 4.4951 | 4.807 | -0.3119 | 416 |
| JB06 | 34.76 | 4.4589 | 4.197 | 0.2619 | 346 |
| JB07 | 2.674 | 5.5728 | 5.527 | 0.0458 | 329 |
| JB08 | 4.165 | 5.3804 | 5.4 | -0.0196 | 500 |
| JB09 | 11.16 | 4.9523 | 5.201 | -0.2487 | 388 |
| JB10 | 5.696 | 5.2444 | 5.134 | 0.1104 | 402 |
| JB11 | 4.704 | 5.3275 | 5.292 | 0.0355 | 450 |
| JB12 | 15.78 | 4.8019 | 4.584 | 0.2179 | 362 |
| JB13 | 10.39 | 4.9834 | 4.863 | 0.1204 | 446 |
| JB14 | 18.62 | 4.73 | 4.974 | -0.244 | 474 |
| JB15 | 0.552 | 6.2581 | 6.244 | 0.0141 | 570 |

^a^pIC_50_ = -log_10_(IC_50_).
