# Supplementary material for: 19-(Benzyloxy)-19-oxojolkinolide B (19-BJB), an ent-abietane diterpene diepoxide, inhibits the growth of bladder cancer T24 cells through DNA damage
Source: PLoS One. 2021 Mar 16;16(3):e0248468. doi: 10.1371/journal.pone.0248468 (PMC7963099; doi:10.1371/journal.pone.0248468)
Supplement: S1 File — (PDF) [file pone.0248468.s010.pdf]

中國醫藥大學實驗動物照護及使用委員會審查同意書

Affidavit of Approval of Animal Use Protocol
China Medical University

動物實驗申請表編號：2016-238

計畫申請人：張永俊　職稱：助理研究員
單位：中草藥研究中心　飼養/應用地點：癌症大樓動物中心
計畫名稱：由天然物骨架設計合成酪氨酸激酶家族 c-Ret、c-Kit、c-Abl 之蛋白抑制劑

本計劃之「動物實驗計畫書」業經實驗動物照護及使用委員會
☑實質 □形式審查通過。本計畫預定飼養應用之動物如下：

動物種類及品系	動物數量	飼養及應用期間
NOD SCID	120	2016/02/01~2016/12/31
NOD SCID	180	2017/01/01~2017/12/31
NOD SCID	180	2018/01/01~2018/07/31

The animal use protocol listed below has been reviewed and approved by the Institutional Animal Care and Use Committee (IACUC)

Protocol Title： Design and synthesis of tyrosine kinases c-Ret, c-Kit, and c-Abl inhibitor from natural products skeleton
Protocol No：2016-238
Period of Protocol： Valid From： 02/2016　To：07/2018　(mm/yyyy)
Principle Investigator (PI)： Jang, Yeong-Jiunn

實驗動物照護及使用委員會召集人：實驗動物照護及使用委員會召集人吳啓瑞　日期：2016.02.15

IACUC Chairman： Chi-Rei Wu　Date：

中國醫藥大學動物實驗申請表

由實驗動物照護及使用委員會填寫(IACUC Use Only)	動審表編號：2016-238
收件日期：	執行期限：105/8-107/7
核准日期：	

一、基本資料

計畫主持人姓名：張永俊	辦公室電話：04-2205-2121#7832
單位：中草藥研究中心	行動電話：0921-004-826
職稱：助理研究員	電子信箱：jinrain.tw@gmail.com
聯絡人姓名:楊顥丞	聯絡電話 :04-2205-2121#7832
計畫名稱(中文):由天然物骨架設計合成酪氨酸激酶家族c-Ret、c-Kit、c-Abl之蛋白抑制劑<br>Project Title(English)：Design and synthesis of tyrosine kinases c-Ret, c-Kit, and c-Abl inhibitor from natural products skeleton	
計畫種類：<br>☐醫學研究 ☐教學訓練 ■藥物及疫苗<br>☐農業研究 ☐健康食品 ☐其他：	
申請類別：<br>■新計畫 ☐延續計畫（原動審表編號：　　　　）	
經費來源：科技部	
計畫執行期限自：02/2016　至：07/2018　(mm/yyyy)	
動物飼養期限自: 02/2016　至：07/2018　(mm/yyyy)	

二、負責進行動物實驗之相關人員資料

姓名	職稱	電話	參與動物實驗年數/教育與訓練經歷[a]	是否已參加本校動物中心使用訓練課程
楊顥丞	博士後研究員	附醫 7832	高雄醫學大學動物中心訓練	■是 ☐否
徐瑋佑	研究助理	附醫 7832	由楊顥丞指導	■是 ☐否
姚宗佑	研究助理	附醫 7832	由楊顥丞指導	■是 ☐否

戴昀皓	研究助理	附醫 7832	由楊顓丞指導	■是 □否

[a] 參與動物實驗年數/教育與訓練經歷：請填寫在本校或其他單位之訓練經歷，無經驗者請填寫由 XXX 老師指導

三、實驗所需之動物（請詳實填寫，不同種類及品系請分別列出，執行多年期計畫者，請分年度列述）：

年度	動物別[a]	品系	使用量	年齡/性別	來源[b]	飼養場所	是否需要繁殖[C]
2016	Mice	NOD SCID	120	4 周/Male	樂斯科	癌症中心大樓動物房	否
2017	Mice	NOD SCID	180	4 周/Male	樂斯科	癌症中心大樓動物房	否
2018	Mice	NOD SCID	180	4 周/Male	樂斯科	癌症中心大樓動物房	否

[a]：保育類野生動物請加註，並另依野生動物保育法相關規定辦理。

[b]：自野外捕捉之動物請加註，並另說明來源地區、隔離檢疫方式及隔離期間；取自民間市場者，必要時須比照辦理。

[b]：動物來源請明確填寫: 國家實驗動物中心、樂斯科、XX 大學實驗動物中心、民間飼養場(場名)、其他(詳細填寫)。

[C]：如需繁殖「實驗動物(指供作科學應用目的使用者)」，請填寫附錄一。

四、動物飼養場所

■本校動物中心

□本校非動物中心飼養場所：＿＿＿＿＿＿＿＿＿＿，

請說明飼養環境，如：溫度、濕度、飼料、飲水、光週期與墊料。

□其他寄養場所：＿＿＿＿＿＿＿＿＿＿

請說明飼養場所之設備、飼養管理措施、負責人及聯絡電話，及原則上須提供該場所經核准營業之證明文件。

五、動物飼養管理

■由實驗動物中心代養　　□由寄養場所負責

□由實驗室人員自養

如由實驗室人員負責，請說明其對動物飼養之背景與訓練。

六、說明動物實驗／操作場所

■實驗動物中心

□個人實驗室或其他地方＿＿＿＿＿＿＿＿＿＿

七、請簡述本研究之目的：

評估由天然物骨架設計合成酪氨酸激酶之蛋白抑制劑對於動物毒性以及抑制動物體內腫瘤生長之能力。

八、請以動物實驗應用 3Rs 之替代及減量原則，說明動物實驗設計、實驗動物需求、動物種別及數量之必要性：

（一）活體動物試驗之必要性，以及選擇此動物種別的原因：

測試藥物皆已經由體外細胞實驗，初步了解藥物毒殺癌細胞之作用與機轉，但細胞試驗無法完全滿足所需資訊，對活體動物之毒性以及抑制腫瘤生長之效果，仍須經由動物實驗確效，以評估後續開發潛力，所以動物實驗有其必要性。選擇 mice 為對象，因其體積小，品系固定，樣本間差異較低，可以最少的動物數目得到可信的結果。

（二）法源依據：

■動物保護法　　■藥品非臨床試驗安全性規範

□健康食品安全性評估方法　　□中華藥典(USP)

□其他＿＿＿＿＿＿＿＿＿＿

（三）參考文獻：（進行此實驗所需動物模式、種類、數量及實驗方法等所參考之國內外期刊論文）

Synergistic Interaction between the HDAC Inhibitor, MPT0E028, and Sorafenib in Liver Cancer Cells In Vitro and In Vivo. ***Clin Cancer Res***. 2014: doi: 10.1158/1078-0432.CCR-12-3909

Tunicamycin potentiates cisplatin anticancer efficacy through the DPAGT1/Akt/ABCG2 pathway in mouse Xenograft models of human hepatocellular carcinoma. ***Mol Cancer Ther***. 2013; 12(12):2874-84.

（四）請儘可能依照統計分析方法說明動物實驗設計(動物分組方法、每組使用動物數量等)：

第一年

MTD 實驗，將 NOD SCID 分為 4 組，給予 vehicle 和低、中、高三種不同劑量的測試藥物，每組各 6 隻，將測試 5 種藥物，因此需要 NOD SCID mice 120 隻

(4x6x5=120)。

第二年

Xenograft 腫瘤實驗，將 NOD SCID 分為 5 組，分別是對照組、正對照組以及三種不同給藥劑量或頻率的給藥組，每組各 7 隻。動物建立腫瘤的成功率為八成，故一次實驗需要 45 隻動物((5x7)/80%=45)。本計畫預計測試 2 種化合物，並將測試兩種不同特性之肝癌細胞株，所以需要 nude mice 180 隻(45x2x2=180)。

第三年

Xenograft 腫瘤實驗，將 NOD SCID 分為 5 組，分別是對照組、正對照組以及三種不同給藥劑量或頻率的給藥組，每組各 7 隻。動物建立腫瘤的成功率為八成，故一次實驗需要 45 隻動物((5x7)/80%=45)。本計畫預計測試 2 種化合物，並將測試兩種不同特性之肝癌細胞株，所以需要 nude mice 180 隻(45x2x2=180)。

九、請以實驗動物應用 3Rs 之精緻化原則，說明實驗中所進行之動物實驗內容：

請詳細說明實驗中所進行之動物實驗內容、方法、劑量與步驟（含動物保定、投藥、注射、麻醉、手術及術後照顧等，無須敘述動物犧牲後檢體之實驗分析方法)，並簡述使動物痛苦降至最低的方法（執行多年期計畫者，若動物實驗內容不同，請分年度列述)。

(一) 動物之保定、禁食、禁水、限制行動（如代謝籠、跑步機、行為實驗）的方法及時間：

植入腫瘤細胞與給藥時，實驗人員短時間徒手對動物進行保定。

並無對動物進行其他禁水，禁食或限制行動。

(二) 簡述整個實驗流程與內容，包括投予何種物質(如藥物、細胞株、感染性物質等)、劑量、方式(靜脈、皮下、腹腔注射等)與頻率。

MTD 實驗

1. 由樂斯科購得 4 週齡 NOD SCID mice，於動物中心代養並適應一週
2. 將 NOD SCID mice 依體重分為四組，每組各六隻:

   A 組(對照組):依給藥組給藥途徑，每天給予 vehicle，連續七天

   BCD 組(給藥組):分為低中高三種不同劑量，每天經口服或腹腔注射投藥一次，連續七天
3. 由給藥開始每天測量體重並仔細觀察動物，持續觀察 21 天後結束實驗。

Xenograft 腫瘤實驗

1. 由樂斯科購得 4 週齡 NOD SCID mice，於動物中心代養並適應一週
2. 在 NOD SCID mice 背部皮下打入肝癌細胞($1x10^7$ cells/0.2ml)
3. 約三週後，將成功建立腫瘤的動物分成五組並秤重
4. A 組(對照組):依給藥組的給藥途徑和頻率給予 vehicle

   B 組(正對照組):每四天由靜脈注射給予 taxol 20mg/kg 一次，共五次

   CDE 組(給藥組):分為三種不同劑量或給藥頻率，每天經靜脈注射或腹腔注射投藥一次
5. 每周兩次測量腫瘤大小及老鼠體重
6. 以腫瘤 $1500mm^3$ 為人道終點或是 60 天結束實驗，利用 CO_2 將動物犠牲

（三）有無進行外科手術？■無；□有，若有請填寫下列事項：

□非存活手術(Non-Survival Surgery) ____________

□存活手術(Survival Surgery) ____________

（四）若實驗含外科程序，請簡述麻醉方法、劑量、投藥方式與手術後的照顧：

（1）麻醉前處理：□動物禁食 □動物不禁食 □其他____________

（2）麻醉前給藥：□需 □不需 □其他 ____________

（3）麻醉方法及麻醉劑

麻醉方法	吸入性麻醉	注射性麻醉(請填寫劑量及注射方法)[a]
麻醉劑	□ $CO_2+10\sim50\%\ O_2$<br>□ Halothane<br>□ Isoflurane<br>□ 其他	□ Pentobarbital[b] ____________<br>□ 已有管制藥品登記證，證號________<br>□ 已申請，審核中<br>□ 尚未申請<br>□ Ketamine[b] ____________<br>□ 已有管制藥品登記證，證號________<br>□ 已申請，審核中<br>□ 尚未申請<br>□ Ketamine ＋Xylazine ____________<br>□ Zoletil(舒泰)____________<br>□ Zoletil＋Xylazine ____________<br>□ Urethane(限用於動物非存活實驗之麻醉)<br>□ 其他____________

[a]：注射方法：IV(靜脈注射)，IM(肌肉注射)，SC(皮下注射)，IP(腹腔注射)。

[b]：Pentobarbital 和 Ketamine 為第三級管制藥品，須先取得管制藥品登記證後方能使用

（五）術後照顧及如何使動物之緊迫或疼痛降至最低(例如使用鎮靜劑或止痛劑、添加環境豐富化物件等)（請參考實驗動物組網頁>動物實驗參考資料>附表7.8(評估與舒緩實驗動物之疼痛）（以下欄位請勿留白）

(1) 依疼痛標準級別與實驗目的，請描述動物疼痛處理方式

疼痛等級	處置方式
□重度疼痛<br>(例如：複雜腹腔切開/器官摘除手術、胸腔切開術、脊髓損傷、傷燙傷、大面積皮膚創傷等)	□傷口照護：方式________<br>□止痛劑：名稱______ 劑量______<br>投予方式______ 頻率______<br>□抗生素：名稱______ 劑量______<br>投予方式______ 頻率______<br>□其他：________
□中度疼痛<br>(例如：腹腔切開手術、卵巢切除術等)	□傷口照護：方式________<br>□止痛劑：名稱______ 劑量______<br>投予方式______ 頻率______<br>□抗生素：名稱______ 劑量______<br>投予方式______ 頻率______<br>□其他：________
■輕度疼痛<br>(例如：剪尾、打耳標/號、植入導管、植入腦部電極、體表植入腫瘤、眼窩靜脈叢採血、皮內注射抗原、睪丸摘除等)	■傷口照護：方式 給予消毒、按壓方式止血。<br>□其他：________
□實驗處理不將使動物產生疼痛反應<br>(例如:單純繁殖動物、衛兵鼠、老年動物安養等)	

(2) 添加環境豐富化物件__________

（六）實驗預期結束之時機，以及動物出現何種異常與痛苦症狀時提前人道終止實驗(humane endpoints):

依據動物保護法第三章第十七條：「科學應用後，應立即檢視實驗動物之狀況，如其已失去部份肢體器官或仍持續承受痛苦而足以影響其生存品質者，應立即以產生最少痛苦之方式宰殺之。」

如安樂死確實影響實驗結果，死亡為唯一實驗終結點，研究人員應在動物實驗申請表中敘述原因或舉出科學例證，並經 IACUC 審核同意。

(1)非腫瘤試驗，請詳閱(表 1)後填寫

(表 1). 安樂死時機與準則如下表:

若此實驗動物出現以下症狀，即為 humane endpoints，將立即安樂死該實驗動物。

體重下降：快速失去原體重的 15-20%、或成長期動物持續無增重、未監測體重但動物呈現惡病質及持續性肌肉消耗時。
食慾喪失或虛弱：動物非鎮靜或麻醉狀況下，無法自行攝食及飲水。小型囓齒類動物於 24-36 小時、大型動物於 5 天完全不進食時，或者小型囓齒類動物於 3 天、大型動物於 7 天僅攝食少量食物時（低於 50%正常攝食量）。
感染：呈現體溫上升或異常的血檢數值，對藥物治療無良好反應且持續演變為全身性不適症狀出現時。
器官臟器的失能，對治療無反應，或由動物中心獸醫師評估為預後極差者，如：<br>➢ 呼吸道系統：嚴重呼吸道感染、呼吸困難。<br>➢ 循環系統：嚴重貧血、無法控制的出血現象、(PVC 低於 15%)、黃疸。<br>➢ 消化道系統：疾病或實驗造成嚴重持續性嘔吐或下痢超過 3 天、消化道阻塞、腹膜炎、腹圍擴大。<br>➢ 泌尿生殖系統：腎衰竭、腹腔積尿。<br>➢ 肌肉骨骼系統：肌肉損傷、骨骼受損、四肢無法行走。<br>➢ 神經系統：異常的中樞神經反應（抽搐、顫抖、癱瘓、歪頭等)、無法有效控制疼痛。<br>➢ 皮膚：持續性的自殘行為、不癒合的傷口、嚴重皮膚炎超過 10%體表面積。

■已詳閱以上內容並同意遵行準則。

☐ 因實驗內容需求，無法配合上述安樂死準則。

請詳填原因:______________________________

請詳填參考文獻:__________________________

(2) 腫瘤試驗，請詳閱(表 1)與(表 2)後填寫

(表 2). 腫瘤試驗之動物安樂死時機與準則:

除上列安樂死準則外，若接種腫瘤動物出現以下臨床症狀時，即為 humane endpoints，將立即安樂死該實驗動物。

平均腫瘤直徑在小鼠超過 20mm、在大鼠超過 40mm。
腫瘤生長超過動物原體重的 10%。
腫瘤轉移。
腫瘤潰爛，造成感染或壞死時。

■已詳閱以上內容並同意遵行準則。

□ 因實驗內容需求，無法配合上述安樂死準則。

請詳填原因:________________________________

請詳填參考文獻:__________________

（七）獲取多株抗體之動物實驗？■無；□有，若有請填寫下列事項：

(1) 使用抗原之全名

(2) 採血所使用之保定方法

(3) 採血的方式與頻率

十、實驗結束後動物處置方法（如復原處置、安樂死、屍體處理方法、轉讓...等；若為轉讓，請提供計畫實驗申請書）：

(1)處置方法：□復原處置 ___________

□轉讓給 ___________

■安樂死

(2)安樂死方法：

□麻醉後頸椎脫臼，麻醉劑：__________________	
□麻醉後斷頭，麻醉劑：__________________	
□麻醉後採血或放血致死，麻醉劑：__________________	
■CO_2	□深度麻醉中灌流，麻醉劑：__________________
□Pentobarbital overdose, Dose (mg/Kg)：___________，給予方式：□靜脈□腹腔	
□其他:____	

(3)屍體處理方法：

■包裝好冰存在動物中心2樓屍體冷凍庫，統一交由感染性廢棄物廠商焚化處理

□其他:______________________________________

十一、有無進行危險性實驗，如生物危險(含感染性物質、致癌藥物)、放射線及化學危險（含毒物）實驗？ ■ 無 □有

如有，請填寫下列事項

1. 實驗之危險性屬於 □生物危險 □放射線 □毒性化學危險

   (A) 進行危險物品之名稱 ________________、實驗方法、途徑及實驗地點

   (B) 說明針對實驗人員、實驗動物以及周邊人畜環境可能之危害，及所採行之保護措施

   (C) 實驗廢棄物與屍體之處理方式

2. 如屬生物危險實驗，
   詳述危害物質生物安全等級 ________________
   是否已送生物安全委員會審核 是□ 否□

3. 如屬放射線或毒性化學危險實驗，請說明本案向主管機關之申請狀況：
   （放射線物質實驗須經行政院原子能委員會認可；毒性化學實驗須經行政院環境保護署認可。）

   □ 尚未申請。

   □ 已申請，審核中。

   □ 通過認可，

   使用危險物質之認可證件名稱與證號________________

   使用危險物質人員之認可證件名稱與證號________________

   實驗地點 ________________

我保證以上所填資料完全屬實

並確認此申請案之執行與運作符合「動物保護法」及相關法規之規定

(若有申請補助計畫(科技部)需檢附「申請動物實驗倫理 3R 說明」時，請填寫附錄二)

申請人簽章 張永俊

申　請日期 104.12.17

初審結果

☑ 初審通過

☐ 改善後再審

☐ 不通過

須改善或不通過之審查意見：

評審人簽章 ______________ 日期 1050202

複審結果

☑ 照案通過

☐ 改善後複審

☐ 不通過

須改善或不通過之審查意見：

中國醫藥大學 105. 2. -5 實驗動物照護及使用委員會

評審人簽章 ______________ 日期 ______________

最終審查結果

☑ 照案通過

☐ 不通過

實驗動物照護及使用委員會召集人簽章 實驗動物照護及使用委員會召集人吳啓瑞 ______________ 日期 2016 0215

附錄一(如有繁殖實驗動物時，請填寫本表。)

實驗動物繁殖表

一、請說明本研究計畫須繁殖動物的理由：

二、列舉所有需繁殖的動物品種與品系、數量等：

繁殖動物總量：		使用於實驗的子代數量
品種/品系：		
種原數量：	子代數量：	
品種/品系：		
種原數量：	子代數量：	
品種/品系：		
種原數量：	子代數量：	

三、動物繁殖之負責單位：

☐ 由動物中心專人負責。

☐ 由實驗室人員負責，請說明其對動物飼養之背景與訓練：

四、請說明種原動物與子代的淘汰策略：

五、未使用於實驗的動物之處置方法：

☐種原：

☐子代：

六、是否為基因改造動物?

☐否

☐是：請填寫下列問題：

(一) 本基因改造動物之來源

☐國家實驗動物中心實驗鼠種源庫

☐購自國外，來源 ____________

☐其他研究人員分讓，來源 ____________

☐自行開發改造。請回答以下問題

1.本實驗進行實驗動物改造之理由

☐經查詢國家實驗動物中心實驗鼠種源庫，無符合需求之基因改造動物

☐相關基因改造動物難以取得或過於昂貴，必須自行開發

☐其他 ____________

2.本實驗基因改造動物開發完成後如何進行保種與分享

☐使用國家實驗動物中心實驗鼠種源庫服務

☐其他種源管理單位____________

☐不保種或不分享，理由____________

(二)請說明動物是否有任何特殊表現型或先天性異常?

☐否

☐是：請說明：____________________________

(三)是否需特殊照養?

☐否

☐是：請說明：____________________________

(四)請說明篩選基因用採樣方法與採樣時間：

計畫申請人簽名：張永偉

日期：104.12.17

附錄二(若有申請補助計畫需檢附 3R 說明時，請填寫本說明。)

動物實驗人道管理替代、減量及精緻化(3R)說明

本研究計畫涉及動物實驗，已考量「替代（Replace）」、「減量（Reduce）」及「精緻化（Refine）」之 3R 精神，將實驗設計最佳化，並說明如下：

一、3R 原則：(複選)

■本實驗計畫已經本人及「中國醫藥大學實驗動物照護及使用委員會」詳實審查，無其他替代方案。

■本實驗計畫已經本人及「中國醫藥大學實驗動物照護及使用委員會」詳實審查，已使用最少數量動物。

■本實驗計畫已經本人及「中國醫藥大學實驗動物照護及使用委員會」詳實審查，已做到精緻化，或動物福利最佳化。

包含：

■已考慮並要求執行動物疼痛評估

■已考慮並要求執行適當減輕動物痛苦方式（如：□麻醉劑、□止痛劑、■設定人道安樂死時機）

□其他(請說明)：____________________

二、教育訓練：(複選)

為促進 3R 精神之落實，本研究實際負責進行動物實驗之相關人員之教育與訓練經歷：

□農委會實驗動物人道管理(例如：動物福利、3R 原則)

■實驗專業技術訓練

□其他(請說明) ：____________________

三、使用動物來源：

為確保本研究計畫實驗品質與效益，本實驗之動物來源為：

■ AAALAC 認證繁殖機構：

■國家實驗動物中心■樂斯科□其他____________

□其他繁殖機構＿＿＿＿＿＿＿＿＿(請註明名稱及地址等)
□其他（請說明）

＿＿＿＿＿＿＿＿＿＿＿＿＿＿＿＿＿＿＿＿＿＿＿＿＿＿＿＿

四、實驗動物飼養環境：

■於本校實驗動物中心飼養，飼養環境符合規範

□校內實驗室＿＿＿＿＿＿

□於校外合格單位飼養，單位＿＿＿＿＿＿

□不飼養，操作後立即結束實驗

五、監督機制：

為確保實驗品質與效益，本研究計畫相關動物實驗之監督機制為：

■「實驗動物照護及使用委員會」，隸屬機構層級＿校級＿＿

■召集人職稱＿中國藥學暨中藥資源學系副教授＿＿

■已設置專責專職獸醫師，並參與計畫審查及動物照護與管理

□計畫審查已包括外部委員

六、行政院農業委員會最近一次實地查核本機構「動物科學應用」之評比紀錄：

□優、□良、■尚可、□較差，查核年度：＿103＿年（請附相關公文書）

七、若行政院農業委員會最近一次實地查核本機構「動物科學應用」之評比為「較差」，建議改善事項之改善情形說明如下：

＿＿＿＿＿＿＿＿＿＿＿＿＿＿＿＿＿＿＿＿＿＿＿＿＿＿＿＿

＿＿＿＿＿＿＿＿＿＿＿＿＿＿＿＿＿＿＿＿＿＿＿＿（請附佐證資料）

計畫申請人簽名：張永信

日期：104.12.17

動物實驗申請表之計畫摘要及動物實驗內容申請書

編號 (由本委員會填寫)：	收件日期：

計畫名稱：由天然物骨架設計合成酪氨酸激酶家族 c-Ret、c-Kit、c-Abl 之蛋白抑制劑		
執行期限：2016/02/01~2018/07/31		
主持人：張永俊	單位：中草藥研究中心	電子信箱：jinrain.tw@gmail.com
聯絡人：楊顓丞	電話：04-2205-2121#7832	電子信箱：q9113054@yahoo.com.tw

動物品系及隻數	第一年：NOD SCID 120 隻。<br>第二年：NOD SCID 180 隻。<br>第三年：NOD SCID 180 隻。
計劃種類	藥物與疫苗
實驗性質	MTD 實驗與 Xenograft 腫瘤實驗
麻醉藥種類劑量	無
止痛劑種類劑量	無
人道終止點	動物若有腫瘤大小超過 1500mm3、體重減輕超過 20%、虛弱而無法進食、感染……等情形發生
安樂死方法	利用 CO2 對動物進行安樂死
生物安全危險性實驗	無
動物繁殖	無

計畫摘要:

The aim of this proposal focuses on developing hinokiflavone-based tyrosine kinase inhibitors (TKIs) for cancer therapy. In this project, we will synthesize the series compounds based on the hinokiflavone structure, and investigate the similar pharmacological inhibitor of ABL1 kinase. We will examine the ABL1 inhibitory affects of hinokiflavone-based derivatives using cell viability assay. ABL1 kinase is constitutively activated in highly aggressive, invasive breast and liver cancer cells . To determine whether inhibition of ABL1 kinase contributes to the invasiveness of cancer cells, we would examine whether blocking ABL1 activation using the inhibitor, STI571, or treatment of hinokiflavone-based derivatives, affects breast and/or liver cancer invasion. Phosphorylated Abl, and Crk/CrkL blots and immunoprecipitation will be used to assess the effect that hinokiflavone-based derivatives having the capacity to decrease Abl1 phosphorylation. To evaluate the therapeutic effects of hinokiflavone-based compounds, nude mice bearing breast and liver cancer cells xenografts will be treated with our synthesized compounds or STI571 daily for 6 weeks. The survival analysis using Kaplan-Meier survival curve and vice versa by comparing with tumor size would be performed to assess the therapeutic efficacy.

動物實驗內容:

請詳細說明本計畫所進行之動物實驗之目的與步驟，例如手術過程、術後處理、抽血量、給藥總體劑量、分組情形、實驗終點等等(無須敘述動物犧牲後檢體之實驗分析方法)。

MTD 實驗(測試毒性)

1. 由樂斯科購得 4 週齡 NOD SCID mice，於動物中心代養並適應一週
2. 將 NOD SCID mice 依體重分為四組，每組各六隻:
   A 組(對照組):依給藥組給藥途徑，每天給予 vehicle，連續七天
   BCD 組(給藥組):分為低中高三種不同劑量，每天經口服或腹腔注射投藥一次，連續七天
3. 由給藥開始每天測量體重並仔細觀察動物，持續觀察 21 天後結束實驗。

Xenograft 腫瘤實驗(觀察藥物藥效)

1. 由樂斯科購得 4 週齡 NOD SCID mice，於動物中心代養並適應一週
2. 在 NOD SCID mice 背部皮下打入肝癌細胞(1x107 cells/0.2ml)
3. 約三週後，將成功建立腫瘤的動物分成五組並秤重
4. A 組(對照組):依給藥組的給藥途徑和頻率給予 vehicle
   B 組(正對照組):每四天由靜脈注射給予 taxol 20mg/kg 一次，共五次
   CDE 組(給藥組):分為三種不同劑量或給藥頻率，每天經靜脈注射或腹腔注射投藥一次
5. 每周兩次測量腫瘤大小及老鼠體重
6. 以腫瘤 1500mm3 為人道終點或是 60 天結束實驗，利用 CO2 將動物犧牲

中國醫藥大學實驗動物照護及使用委員會

動物實驗申請表送審收件證明

計畫申請人：張永俊　　職稱：助理研究員
單位：中草藥研究中心
計畫名稱：由天然物骨架設計合成酪氨酸激酶家族 c-Ret、c-Kit、c-Abl 之蛋白抑制劑

送件日期：104/12/17

茲證明上述之計畫案，本校實驗動物照護及使用委員會業已收到所送審之動物實驗申請表，目前尚在審查中，特核發此函以茲證明。

計劃主持人簽名：張永俊

實驗動物照護及使用委員會：
