# Supplementary material for: 19-(Benzyloxy)-19-oxojolkinolide B (19-BJB), an ent-abietane diterpene diepoxide, inhibits the growth of bladder cancer T24 cells through DNA damage
Source: PLoS One. 2021 Mar 16;16(3):e0248468. doi: 10.1371/journal.pone.0248468 (PMC7963099; doi:10.1371/journal.pone.0248468)
Supplement: Attachment — Submitted filename: Response to Reviewers.docx [file pone.0248468.s011.docx]

**All line numbers mentioned below correspond to the document “Manuscript”.**

**Editor:**

1. Please describe rationale and cellular phenotypes for the cell lines used in the study, particularly for the chemoresistant ones.

Answer: The rationale and cellular phenotypes for cell lines were described in line 93-100. Most of them were searched from ATCC.

2. Experiments should be performed to find out 19-BJB toxicity in non-malignant cells.

Answer: HaCat cells were chosen to perform the toxicity of 19-BJB. The result was in Fig 2F.

3. More specific inhibitors of checkpoint kinases should be used in the experiments (to avoid caffeine non-specific effects), and similar analyses (WB, fig 5) should be performed with these inhibitors (not only live-dead assay).

Answer: We had changed all experiments about caffeine to KU-55933, an ATM inhibitor (ATMI in manuscript). This drug was purchased from Sigma-Aldrich. The chemical structure is in S5 Fig. The cell cycle test of T24 was also performed. The new experiments about cell cycle, WB and live-dead assay were shown in Fig 5, 8, and 9. The related descriptions in Materials/Methods and Results were also changed. A more detailed discussion was presented in line 541-584.

**Reviewer 1:** It would be interesting to investigate whether the treatment with this class of drugs may affect the DSBs repair by NHEJ or HR. The authors should assess the DSBs repair by utilizing specific reporter assays.

Answer: We had changed the caffeine (non-specific effects agent) to KU-55933, a specific ATM inhibitor to avoid confusing result. In the new experiment of live and dead (Fig 9), we found that 19-BJB had a synergistic effect with ATM inhibitor. The FACS analysis of cell cycle (Fig 5) showed obvious G1/S arrest in 24 h group, and not obvious G2/M arrest in 48 h group. The apoptosis peak became more obvious as the concentration of 19-BJB was increased, especially in the cells after 48 h treated. So we considered that the DNA damage effect of 19-BJB could not be eliminate in 48 h which means a failure of DNA repair. A more detailed discussion was presented in line 541-584.

**Reviewer 2:**

1. There is no demonstration of strand-breaks or adducts. The mechanism of DNA damage needs to be clarified.

Answer: We had rewritten paragraphs about DNA damage in 541-558. Here are two reasons that make us think about DSBs (double-strand breaks) effect of 19-BJB. Firstly, ATM plays an important role in DNA damage, especially after DSBs. ATM is recruited to the sites of DSBs by sensor protein. Then the signals are transduced to downstream effectors such as Chk2/p-H2AX/p53, leading to cell cycle arrest [44]. Secondly, the presence of p-H2AX is the direct evidence of DBS in DNA [51]. In Fig 7, the expression of p-Chk2 and p-H2AX increased which showed a DSBs may occur.

2. The data obtained with cell lines also does not prove the conclusion. There is a discrepancy between the concentrations required to induce DNA damage and cytotoxicity, respectively. To me, it seems that apoptosis is induced by lower concentrations than DNA damage (Fig. 5). In addition to other experiments, it would be important to establish the time course of events as well.

Answer: To establish the time course of events is a good suggestion. In cell cycle analysis (Fig 5 in new manuscript), we had chosen 24 h and 48 h after treatment as different groups. The apoptosis peak was observed as the concentration of 19-BJB was increased in the cells after 48 h treated, but not so obvious in 24 h group. In Fig 7 of new manuscript (original Fig 5), the evidence of apoptosis is cleaved caspase-3. While cleaved PARP-1, p-Chk1, p-Chk2 and p-H2AX is related to DNA damage. In my opinion, although they have connections, cleaved caspase-3 is considered as the direct evidence. Compared to p-Chk1, p-Chk2 or cleaved PARP-1, the increasing expression of cleaved caspase-3 is not so obvious at low concentration of 19-BJB.

3. Likewise, the binding concentrations in vitro and in vivo do not seem to fit the bill. Have the authors considered intracellular metabolism of the compound? After all, it is an epoxide and activated metabolites could be substantially more reactive.

Answer: It is an interesting question about the metabolites of 19-BJB. Actually, we have considered that if 19-BJB would be decomposed into 19-Acid-JB and BnOH. After all, ester always can be hydrolyzed in cells or tissues. So we had tested the effect of 19-Acid-JB (synthesized by ourselves) and BnOH (considered to have toxicity). Neither of them could inhibit cell growth. The metabolism of epoxide compound could be substantially more reactive, but we cannot synthesize. HPLC-MS may be a good way to solve this problem. The cells should be harvested after treatment with 19-BJB and detected the metabolites in MS after purified in HPLC. The further researches will be considered. Thank you for this suggestion!

4. The analyses of cell death are incomplete. Cell cycle profiles are required. Caffeine is a quite unspecific compound and more specific inhibitors of checkpoint kinases should be used.

Answer: We had changed all experiments about caffeine to KU-55933, an ATM inhibitor (ATMI in manuscript). This drug was purchased from Sigma-Aldrich. The chemical structure is in S5 Fig. The new experiments about cell cycle, WB and live-dead assay were shown in Fig 5, 8, and 9. The related descriptions in Materials/Methods and Results were also changed. A more detailed discussion was presented in line 541-584.

5. It should be explained, why the particular cell lines were used. Important properties of not commonly used cell lines like NT-TUB and its derivatives should be introduced (e.g. p53 status in this context) and they should be referenced. A more representative selection of bladder cancer cell lines, including more differentiated lines, would make sense. Moreover, the tumor-specificity of the treatment needs to be demonstrated, using e.g. fibroblasts and non-transformed urothelial cells.

Answer: The rationale and cellular phenotypes for cell lines were described in line 93-100. Most of them were searched from ATCC. NTUB1, NP14 and NTaxol were chosen because these kinds of bladder cell lines have the most stable drug-resistant effects in our lab. When we finished the synthesis of jolkinolide derivatives to obtain more than 30 kinds of compounds (S1 Fig), it is very important to test the potential of jolkinolide structure about combination medication for the further researches. Cisplatin is commonly used drug in clinical treatment of bladder cancer. Taxol, a natural product same as jolkinolide A and B, also have a good effect in cancer therapy. T24 and J82 are two widely used cell lines which are often used in animal model.

6. The discussion lacks depth and among others, a comparison with findings for other compounds that may have a similar mode of action.

Answer: We had rewritten the paragraph of discussion. The results and detailed discussions of new experiments were presented in line 541-584.

7. For the more biologically experienced reader the significance and limitations of the molecular docking and CoMFA need to be better explained; for chemists and pharmacologists their limitations need to be more critically considered.

Answer: We had rewritten some sentences to make it easier to understand. In this article, QSAR is a highlight that is the first time to explain the detail structure-activity relationship of jolkinolide derivatives, so we kept and revised this part.

8. Some editing for language is required, e.g. phosphor- should read phospho- in l.328, or l.270-272, etc. The introductory statements on bladder cancer are not accurate; I agree that it is an important (and often underestimated) disease, but the incidence has certainly not “skyrocketed” in recent years.

Answer: We had revised the expression mention above and made the manuscript more accurate.
