# Supplementary material for: 19-(Benzyloxy)-19-oxojolkinolide B (19-BJB), an ent-abietane diterpene diepoxide, inhibits the growth of bladder cancer T24 cells through DNA damage
Source: PLoS One. 2021 Mar 16;16(3):e0248468. doi: 10.1371/journal.pone.0248468 (PMC7963099; doi:10.1371/journal.pone.0248468)
Supplement: Attachment — Submitted filename: Response to Reviewers.docx [file pone.0248468.s012.docx]

**All line numbers mentioned below correspond to the document “Manuscript”.**

**Reviewer 1:** Despite the authors have not completely addressed this reviewer requests, the revised manuscript is certainly improved, and is now suitable for publication. Mention to the suggested recommended papers would have been appreciated.

Answer: Thank you for your suggestions before. We had revised the manuscript according to the comments.

**Reviewer 2:** The manuscript is much improved; the data are better explained and the additional experiments have helped to clarify the matter. Several issues remain, however. **Major comments:**

1. I feel that the authors have still not excluded the alternative interpretation of their findings that DNA damage is only part of the mode of action of 19BJB (and perhaps only indirectly so). They observe an increased G1 fraction after 24 h which is followed by apoptosis later. All data on DNA damage response were however obtained at 48 h. At least increased H2A.X phosphorylation should be seen at 24 h. Positive comet assays can also result from (early stages of) apoptosis. 19BJB could therefore act via another mechanism.

Answer: We did not exclude the alternative interpretation, because of the observation of increasing G1 fraction after 24 h. So the expressions of phosphorylation of H2A.X and PARP-1 were checked by western blot after treatment with 19-BJB for 24 h (Line 423-433). The expression of cleaved-PARP-1 was increased in 4 µM and 8 µM groups. The expression of p-H2A.X was also significantly increased in 8 µM group. Taken together, we think that the increasing G1 fraction after 24 h is also caused by DNA damage.

2. This alternative is the more likely as the data (little of which is truly experimental) show binding to DNA and insertion. Thus, the structure of DNA may be disturbed, but does this suffice to elicit a DNA damage response? This should be shown or at least discussed by comparison with other compounds binding to DNA in a similar manner. For instance, bleomycin intercalates (even more deeply) into DNA, but DNA damage is actually caused by a part of the molecule generating hydroxyl radicals. On a related point: how do the authors interpret the change in the UV spectrum? Can this change be explained by insertion and disturbance of pi-pi interactions or would it require unwinding?

Answer: Thank you for your suggestion. At the beginning, we did think that the changes of DNA structure may lead to DNA damage. But bleomycin is a good example to prove that our previous concept was inaccurate. The changes in the UV spectrum are more like “interaction” or “intercalates” but not the evidence of DNA damage.

In this revised manuscript, we checked the expression of 8-oxoguanine (Line 463-477), for it is one of the most common DNA lesions, which can results in a mismatched pairing with adenine resulting in G to T and C to A substitutions in the genome. As the concentration of 19-BJB was increased, the expression of 8-oxoguanine became more significant. This finding indicated that the interaction between 19-BJB and DNA may cause the synthesis of 8-oxoguanine in T24 cells after 24 h. The structural changes of DNA may be the main reason of damage.

**Minor comments:**

3. Another round of language editing is required, especially in the abstract, but also throughout the manuscript.

Answer: We have revised the words in the manuscript and tried to make it be accurate, especially in abstract.

4. Many figures in the pdf are not of sufficient quality. Much better quality needs to be provided in the final version.

Answer: The figures were reorganized to be quality.

5. Throughout the manuscript, including the figures, numbers and units should be separated by a space.

Answer: We have rechecked the manuscript and revised the missing parts again.

6. Throughout the manuscript, round off numbers to a reasonable number of digits. In particular, table 1 would be better legible if corrected in this way.

Answer: We have round off numbers to a reasonable number of digits in table 1 (Line 302-305), but skipped the data of paclitaxel in T24 and NP14 cell lines as the numbers are too small. The other parts of manuscript were also rechecked.

7. The first § on bladder cancer is still inaccurate and the references are not recent or do not fit well.

Answer: We have replaced the references with recent articles and rewritten paragraph 1 in introduction (Line 41-44).

8. Many other statements in the manuscript are also inaccurate, in many cases because the wording is incorrect. Proper scientific terms are especially important. Thus, in cell cycle analysis, “rates” should be replaced by “fractions” (the entire § from l.341 needs correction); chromatin is not “depolymerized” (l.557), “conjugation” is not correct for binding to and insertion into DNA. Please check throughout. l.573f: synergy has not been shown.

Answer: We have changed the words to the accurate expression. For example, “rates” was replaced by “fractions”, “conjugation” was replaced by “interaction”. The sentence related “depolymerized” was modified and “synergy” was deleted, respectively.
